# Supplementary material for: Gut Microbiota and Subclinical Cardiovascular Disease in Patients with Type 2 Diabetes Mellitus
Source: Nutrients. 2021 Aug 1;13(8):2679. doi: 10.3390/nu13082679 (PMC8397936; doi:10.3390/nu13082679)
Supplement: Supplementary file 1 [file nutrients-13-02679-s001.zip › nutrients-1284705-supplementary.pdf]

Supplemental Table S1. The primers used in real-time quantitative PCR.

Target	Primer (5'-3')	References
Phylum: *Firmicutes*	F: GGAGYATGTGGTTTAATTCGAAGCA	[1]
	R: AGCTGACGACAACCATGCAC	
C. leptum group (IV)	F: GTTGACAAAACGGAGGAAGG	[2]
	R: GACGGGCGGTGTGTACAA	
F. prausnitzii	F: AGATGGCCTCGCGTCCGA	[2]
	R: CCGAAGACCTTCTTCCTCC	
Phylum: *Bacteroidetes*	F: GGARCATGTGGTTTAATTCGATGAT	[1]
	R: AGCTGACGACAACCATGCAG	
Bacteroides	F: GTCAGTTGTGAAAGTTTGC	[2]
	R: CAATCGGGAGTTCTTCGTG	
Phylum: *Actinobacteria*		
Bifidobacterium	F: AGGGTTCGATTCTGCTCAG	[2]
	R: CATCCGGCATTACCACCC	
Phylum: *Proteobacteria*		
E. coli	F: CATGCCGCGTGTATGAAGAA	[3]
	R: CGGGTAACGTCAATGAGCAAA	
Phylum:*Verrucomicrobia*		
A. muciniphila	F:CAGCACGTGAAGGTGGGGAC	[4]
	R:CCTTGCGGTTGGCTTCAGAT	

References

1. Guo, X.; Xia, X.; Tang, R.; Wang, K. Real-time PCR quantification of the predominant bacterial divisions in the distal gut of Meishan and Landrace pigs. *Anaerobe* **2008**, *14*, 224-228.
2. Wang, W.; Chen, L.; Zhou, R.; Wang, X.; Song, L.; Huang, S.; Wang, G.; Xia, B. Increased proportions of *Bifidobacterium* and the *Lactobacillus* group and loss of butyrate-producing bacteria in inflammatory bowel disease. *J Clin Microbiol* **2014**, *52*, 398-406.
3. Penders, J.; Thijs, C.; Vink, C.; Stelma, F.F.; Snijders, B.; Kummeling, I.; van den Brandt, P.A.; Stobberingh, E.E. Factors influencing the composition of the intestinal microbiota in early infancy. *Pediatrics* **2006**, *118*, 511-521.
4. Karlsson, C.L.; Onnerfalt, J.; Xu, J.; Molin, G.; Ahrne, S.; Thorngren-Jerneck, K. The microbiota of the gut in preschool children with normal and excessive body weight. *Obesity (Silver Spring)* **2012**, *20*, 2257-2261.

Supplemental Table S2. Correlation between targeted microbiota and left ventricular (LV) structure and function.

	Phylum Firmicutes		Phylum Bacteroidetes		Firmicutes/Bacteroidetes ratio		Bacteroides	
LV Ejection fraction (per 1 %)	β (95% Cl)	*P*	β (95% Cl)	*P*	β (95% Cl)	*P*	β (95% Cl)	*P*
Unadjusted model	4.28(0.05,8.50)	0.04	3.80(0.45,7.15)	0.02	-1.42(-5.29,2.44)	0.46	2.99(-0.22,6.20)	0.06
Age and sex adjusted	4.22(-0.01,8.44)	0.08	3.79(0.44,7.14)	0.08	-1.45(-5.31,2.42)	0.46	2.99(-0.22,6.20)	0.06
Multivariate adjusted model 1	4.58(0.28,8.87)	0.03	4.68(1.17,8.19)	0.009	-2.05(-6.14,2.04)	0.32	3.36(0.06,6.65)	0.04
Multivariate adjusted model 2	3.67(-0.64,7.98)	0.09	3.92(0.24,7.61)	0.03	-1.53(-5.73,2.68)	0.47	2.51(-0.93,5.95)	0.15
LV Fraction shortening (per 1 %)	β (95% Cl)	*P*	β (95% Cl)	*P*	β (95% Cl)	*P*	β (95% Cl)	*P*
Unadjusted model	2.89(-0.51,6.29)	0.09	2.88(0.20,5.56)	0.04	-1.37(-4.46,1.72)	0.38	2.35(-0.22,4.93)	0.07
Age and sex adjusted	2.85(-0.55,6.25)	0.10	2.88(0.19,5.57)	0.04	-1.39(-4.49,1.70)	0.37	2.36(-0.22,4.93)	0.07
Multivariate adjusted model 1	3.12(-0.35,6.59)	0.07	3.63(0.80,6.45)	0.01	-1.96(-5.25,1.32)	0.24	2.71(0.06,5.36)	0.04
Multivariate adjusted model 2	2.42(-1.07,5.91)	0.17	2.99(0.01,5.97)	0.04	-1.51(-4.90,1.88)	0.38	2.01(-0.76,4.79)	0.15
LVMI (per 1 g/m^2)	β (95% Cl)	*P*	β (95% Cl)	*P*	β (95% Cl)	*P*	β (95% Cl)	*P*
Unadjusted model	-14.88(-31.04,1.28)	0.07	-7.08(-20.00,5.85)	0.28	-2.99(-17.74,11.76)	0.69	-11.85(-24.01,0.32)	0.05
Age and sex adjusted	-14.52(-30.38,1.34)	0.07	-6.08(-18.80,6.63)	0.34	-4.02(-18.52,10.48)	0.58	-10.90(-22.90,1.11)	0.07
Multivariate adjusted model 1	-13.37(-29.28,2.53)	0.09	-2.37(-15.56,10.82)	0.72	-8.75(-23.81,6.30)	0.25	-7.68(-19.92,4.56)	0.21
Multivariate adjusted model 2	-13.18(-28.88,2.52)	0.09	-3.68(-17.16,9.80)	0.59	-7.53(-22.76,7.70)	0.33	-9.42(-21.91,3.07)	0.13
LVH	OR (95% Cl)	*P*	OR (95% Cl)	*P*	OR (95% Cl)	*P*	OR (95% Cl)	*P*
Unadjusted model	0.38(0.13-1.15)	0.08	0.52(0.23-1.18)	0.12	1.12(0.43-2.95)	0.82	0.48(0.22-1.05)	0.06
Age and sex adjusted	0.38(0.13-1.16)	0.09	0.53(0.23-1.22)	0.13	1.09(0.41-2.90)	0.86	0.49(0.22-1.08)	0.07
Multivariate adjusted model 1	0.33(0.10,1.11)	0.07	0.61(0.24-1.56)	0.30	0.75(0.25-2.27)	0.60	0.59(0.25-1.40)	0.23
Multivariate adjusted model 2	0.24(0.06-0.95)	0.04	0.46(0.15-1.39)	0.17	0.43(0.16-1.19)	0.10	0.43(0.15-1.19)	0.10

Model 1 was adjusted for sex, age, plus smoke, alcohol, body mass index, hypertension, β-blocker, ACRI/ARB, statin.
Model 2 was adjusted for variables of model 1 plus high-density lipoprotein, log-formed low-density lipoprotein, HbA1C, urine albumin-creatinine ratio, and log-formed triglyceride
Abbreviations: LVMI, left ventricle mass index; LVH, left ventricular hypertrophy

Supplemental Table S3. Correlation between targeted microbiota and left ventricular diastolic function

	Phylum Firmicutes		Phylum Bacteroidetes		Firmicutes/Bacteroidetes ratio		Bacteroides	
E/E′	β (95% Cl)	*P*	β (95% Cl)	*P*	β (95% Cl)	*P*	β (95% Cl)	*P*
Unadjusted model	-0.46(-1.69,0.78)	0.46	-0.96(-1.93,0.00)	0.05	0.87(-0.23,1.96)	0.12	-0.82(-1.73,0.10)	0.08
Age and sex adjusted	-0.54(-1.76,0.68)	0.38	-1.05(-2.00,-0.09)	0.03	0.90(-0.18,1.98)	0.10	-0.89(-1.80,0.01)	0.05
Multivariate adjusted model 1	-0.66(-1.91,0.58)	0.29	-1.07(-2.07,-0.07)	0.03	0.80(-0.34,1.94)	0.16	-0.90(-1.82,0.02)	0.05
Multivariate adjusted model 2	-0.57(-1.86,0.71)	0.38	-1.30(-2.36,-0.23)	0.02	1.07(-0.11,2.24)	0.07	-1.06(-2.03,-0.10)	0.03
E/E′ ≥ median *vs.* E/E′ < median	OR(95%CI)	*P*	OR(95%CI)	*P*	OR(95%CI)	*P*	OR(95%CI)	*P*
Unadjusted model	0.76(0.32-1.84)	0.54	0.48(0.23-0.99)	0.04	2.04(0.91-4.57)	0.08	0.55(0.28-1.08)	0.08
Age and sex adjusted	0.74(0.30-1.78)	0.50	0.45(0.22-0.95)	0.03	2.10(0.93,4.75)	0.07	0.52(0.26-1.03)	0.06
Multivariate adjusted model 1	0.66(0.26-1.67)	0.37	0.43(0.19-0.95)	0.04	2.03(0.85-4.87)	0.11	0.51(0.25-1.05)	0.07
Multivariate adjusted model 2	0.65(0.24-1.74)	0.38	0.33(0.14-0.81)	0.02	2.51(0.96-6.55)	0.06	0.42(0.19-0.93)	0.03
E/A < 1	OR(95%CI)	*P*	OR(95%CI)	*P*	OR(95%CI)	*P*	OR(95%CI)	*P*
Unadjusted model	0.87(0.29-2.60)	0.80	1.15(0.49-2.70)	0.74	0.75(0.29-1.95)	0.55	1.08(0.48-2.43)	0.85
Age and sex adjusted	0.83(0.27-2.56)	0.75	1.16(0.47-2.87)	0.75	0.72(0.27-1.96)	0.52	1.13(0.49-2.62)	0.77
Multivariate adjusted model 1	0.80(0.24-2.71)	0.72	1.22(0.45-3.30)	0.69	0.63(0.20-1.98)	0.43	1.20(0.49-2.91)	0.69
Multivariate adjusted model 2	0.88(0.24-3.24)	0.85	1.41(0.48-4.19)	0.53	0.57(0.17-1.98)	0.38	1.26(0.48-3.26)	0.64

Model 1 was adjusted for sex, age, plus smoke, alcohol, body mass index, hypertension, β-blocker, ACRI/ARB, statin.
Model 2 was adjusted for variables of model 1 plus high-density lipoprotein, log-formed low-density lipoprotein, HbA1C, urine albumin-creatinine ratio, and log-formed triglyceride.
The median of E/E′ was 8.84.

Supplemental Table S4. Correlation between targeted microbiota and left atrium diameter

	Phylum Firmicutes		Phylum Bacteroidetes		Firmicutes/Bacteroidetes ratio		Bacteroides	
LA diameter (per 1 cm)	β (95% Cl)	*P*	β (95% Cl)	*P*	β (95% Cl)	*P*	β (95% Cl)	*P*
Unadjusted model	-0.02(-0.30,0.26)	0.88	-0.21(-0.43,0.01)	0.06	0.25(0.00,0.50)	0.04	-0.29(-0.50,-0.09)	0.006
Age and sex adjusted	-0.02(-0.29,0.26)	0.91	-0.20(-0.41,0.02)	0.08	0.24(-0.01,0.49)	0.06	-0.28(-0.48,-0.08)	0.008
Multivariate adjusted model 1	-0.05(-0.30,0.20)	0.72	-0.18(-0.38,0.03)	0.09	0.19(-0.04,0.42)	0.11	-0.27(-0.45,-0.09)	0.004
Multivariate adjusted model 2	-0.04(-0.30,0.22)	0.77	-0.17(-0.39,0.05)	0.14	0.18(-0.07,0.43)	0.16	-0.27(-0.47,-0.07)	0.008
LA diameter ≥ median *vs.* LA diameter < median	OR (95% Cl)	*P*	OR (95% Cl)	*P*	OR (95% Cl)	*P*	OR (95% Cl)	*P*
Unadjusted model	1.12(0.48-2.59)	0.80	0.48(0.24-0.97)	0.04	2.86(1.57-6.42)	0.01	0.48(0.24-0.97)	0.04
Age and sex adjusted	1.14(0.48-2.70)	0.76	0.49(0.24-1.00)	0.05	2.85(1.25-6.51)	0.01	0.31(0.15-0.65)	0.002
Multivariate adjusted model 1	1.20(0.46-3.13)	0.71	0.53(0.24-1.19)	0.12	2.90(1.10-7.62)	0.03	0.29(0.13-0.65)	0.003
Multivariate adjusted model 2	1.26(0.46-3.46)	0.65	0.54(0.23-1.27)	0.16	3.10(1.08-8.95)	0.04	0.30(0.13-0.70)	0.006

Model 1 was adjusted for sex, age, plus smoke, alcohol, body mass index, hypertension, β-blocker, ACRI/ARB, statin.
Model 2 was adjusted for variables of model 1 plus high-density lipoprotein, log-formed low-density lipoprotein, HbA1C, urine albumin-creatinine ratio, and log-formed triglyceride.
The median of left atrium diameter was 3.74 cm.
